# Prospective evaluation of SeptiFast Multiplex PCR in children with systemic inflammatory response syndrome under antibiotic treatment

**DOI:** 10.1186/s12879-016-1722-9

**Published:** 2016-08-08

**Authors:** Franziska Gies, Eva Tschiedel, Ursula Felderhoff-Müser, Peter-Michael Rath, Joerg Steinmann, Christian Dohna-Schwake

**Affiliations:** 1 Department of Pediatrics I, University of Duisburg-Essen, Hufelandstr. 55, 45122 Essen, Germany; 2 Institute of Medical Microbiology, University of Duisburg-Essen, Hufelandstr. 55, 45122 Essen, Germany; 3 Centre Hospitalier Universitaire Bicêtre, Réanimation Pédiatrique et Médecine Néonatale, Hôpitaux Universitaires Paris-Sud AP-HP, 78, Rue du Général Leclerc, 94275 Le Kremlin-Bicêtre, France

**Keywords:** Systemic Inflammatory Response Syndrome (SIRS), Children, SeptiFast, Blood culture, Antimicrobial treatment

## Abstract

**Background:**

Antimicrobially pre-treated children with systemic inflammation often pose a diagnostic challenge to the physician. We aimed to evaluate the additional use of SeptiFast multiplex polymerase chain reaction (PCR) to identify causative pathogens in children with suspected systemic bacterial or fungal infection.

**Methods:**

Prospective observational study in 39 children with systemic inflammatory response syndrome (SIRS) under empiric antibiotic treatment. Primary outcome was the rate of positive blood cultures (BC), compared to the rate of positive SeptiFast (SF) results.

**Results:**

In total, 14 SF-samples yielded positive results, compared to 4 positive BC (*p* < 0.05). All blood cultures and 13 of 14 positive SF-tests were considered infection. Median time for positive BC was 2 days, and time to definite result was 6 days, compared to 12 h for SF. Antimicrobial therapy was adapted in 7 of the 14 patients with positive SeptiFast, and in 3 of the 4 patients with positive BC.

Best predictive power for positive SF shown by receiver-operating characteristic was demonstrated for procalcitonin PCT (Area under the curve AUC: 0.79), compared to C-reactive protein CRP (AUC: 0.51) and leukocyte count (AUC: 0.46). A procalcitonin threshold of 0.89 ng/ml yielded a sensitivity of 0.82 and a specifity of 0.7. Children with a positive SeptiFast result on day 0 had a significantly higher risk to require treatment on the Pediatric Intensive Care Unit or to be deceased on day 30 (Odds-Ratio 8.62 (CI 1.44-51.72).

**Conclusions:**

The additional testing with SeptiFast in antimicrobially pre-treated children with systemic inflammation enhances the rate of pathogen detection. The influence of multiplex PCR on clinically relevant outcome parameters has to be further evaluated. (Trial registration: DRKS00004694)

## Background

In suspected serious bacterial or fungal infection, particularly sepsis, antimicrobial treatment is commonly initiated empirically. If necessary, therapy is modified according to microbial results. Today, blood cultures are considered diagnostic gold standard in sepsis to identify pathogens and to test possible resistance. Unfortunately, gram stainings are usually available earliest after 12–15 h, and definitive results of culture not before 48 h. As time is crucial in sepsis, this delay in diagnostics and hence correct treatment might have detrimental consequences for the patient [1–4].

Additionally, often neither bacteria nor fungus can be found in clinically suspected sepsis in children. To produce reliable results in blood cultures, a minimum number of viable pathogens are required. If the patient is already under antimicrobial treatment, the quantity of circulating pathogens is substantially reduced. And medical staff often faces difficulties in obtaining sufficient amounts of blood in small infants, which is another factor potentially contributing to failure of blood culture [5].

Differentiation between a Systemic Inflammatory Response Syndrome (SIRS) due to an infection and SIRS due to other reasons can become a true challenge. Chronically ill children are often hospitalized for long periods of time. They are prone to SIRS caused by e. g. viral infections, the underlying disease itself, subileus or ileus, or rejection after transplantation. Again, delayed or wrong diagnosis might be detrimental for the patient. Therefore, much hope is being put into newer diagnostic technologies to improve detection rates of infectious pathogens or to exclude bacterial/fungal infections.

SeptiFast is a multiplex polymerase chain reaction (PCR) based method, able to identify desoxyribonucleic acid (DNA) of 25 different bacteria and fungi. Major advantage of this method is the fact that for PCR only very small amounts of pathogens are needed. And, as it is not dependent on growth of bacteria no viable material is needed which means that also killed bacteria can still be detected. Causative pathogens can be detected even under antimicrobial therapy, when viable bacteria or fungi are not present for cultivation anymore.

Based on these considerations, we initialized a prospective observational study in children with systemic inflammation and under antimicrobial treatment. We compared the rates of successful pathogen identification by BC to those by SeptiFast. The primary goal of the study was the rate of positive SF tests compared to the rate of positive BC.

## Methods

Patients: Infants, children, and adolescents were included in this prospective observational study, if they were clinically diagnosed with Systemic Inflammatory Response Syndrome (SIRS) and pre-treated with systemic antibiotics. Time threshold for pre-treatment was either more than 48 h of antibiotic therapy or at least two doses, if the patient was admitted to the pediatric intensive care unit (PICU). This second inclusion criterion was chosen, because it was assumed that a sicker patient would possibly benefit from early diagnosis. SIRS was defined using the International Pediatric Sepsis Consensus Conference criteria from 2005 [6]. The only exclusion criteria were prematurity of 37 weeks or less and age below 28 days.

Outcome criteria: Primary outcome criterion was the rate of positive BC compared to the rate of positive SF results.

Secondary outcome parameters were time to definite result of BC and SF, number of antimicrobial therapy changes according to BC and SF results, and the predictive power for positive SF of additional biomarkers such as C-reactive protein, leukocyte count and procalcitonin. Additionally, we documented the treatment status at day 30 according to the following categories: 1) death, 2) still on pediatric intensive care unit, 3) transferred to or still staying on peripheral ward, 4) discharged from hospital.

Study protocol: In children with SIRS and antibiotic pre-treatment, who were enrolled in the study, personal data including duration of antibiotic therapy and its modification were documented. Vital parameters (heart rate, respiratory rate, body temperature) and infection biomarkers from blood (C-reactive protein CRP, leukocyte count and procalcitonin PCT) were recorded on day 0, day 4-6 and day 30, if available.

Antibiotic treatment, number, and site of BC taken (central versus peripheral) as well as the amount of blood drawn for blood cultures were left to the decision of the attending physician. A manual with advices for optimum BC diagnostics was handed to all participating physicians. The physician in charge assessed all positive BC and SF results for infection, contamination, or whether they were not assessable (n/a). There was no treatment algorithm for a positive SF or BC result provided.

Blood sampling: Blood samples were taken from a peripheral vein or from a central venous line. For SF, at least 1.5 ml blood was collected and charged with ethylendiaminetetraacetic acid. Blood culture bottles were inoculated with 1–10 ml blood, depending on the age of the child and on the type of bottle used (aerobic/anaerobic or both). If possible, paired samples were taken for SF and BC, if not, a second sample was taken as soon as possible not exceeding a delay of 24 h. In all patients, a single SF sample was taken.

Blood culture sample: Depending on the age of the patient, samples were inoculated into Bactec Peds Plus/F (children younger than 36 months) or Bactec Plus/F aerobic and anaerobic bottles (Bactec, Becton Dickinson GmbH, Germany). BCs were incubated for up to 5 days at 36 °C. In case of a positive blood culture result, aliquots were taken for subculture on solid media, Gram-stain and susceptibility-testing via MicroScan WalkAway (Siemens Healthcare Diagnostics, Germany).

Real-Time multiplex PCR: The SeptiFast real-time PCR is capable of the amplification and detection of 25 microorganisms (see Table 1, SeptiFast Masterlist) by using the internal transcribed spacer region of the microorganisms as specific target. A detailed description of the assay has been given elsewhere [7]. Briefly, the assay follows three steps: 1) specimen preparation by mechanical lysis and purification of (DNA), 2) Real-time PCR amplification of target DNA in three parallel reactions (Gram positive, Gram negative, fungi) and subsequent detection of PCR products by specific hybridization probes, and 3) automated identification of species and controls [7].

**Table 1:** SeptiFast Master List (Lehmann 2008)

Gram negative	Gram positive	Fungi
*Escherichia coli*	*Staphylococcus aureus*	*Candida albicans*
*Klebsiella (pneumoniae/oxytoca)*	*Coagulase-negative staphylococci*	*Candida tropicalis*
*Serratia marcescens*	*Streptococcus pneumoniae*	*Candida parapsilosis*
*Enterobacter (cloacae/ aerogenes)*	*Streptococcus species*	*Candida krusei*
*Proteus mirabilis*	*Enterococcus faecium*	*Candida glabrata*
*Pseudomonas aeruginosa*	*Enterococcus faecalis*	*Aspergillus fumigatus*
*Acinetobacter baumanii*		
*Stenotrophomonas maltophilia*		

Coagulase-negative staphylococci (*S. epidermidis, S. haemolyticus*)

Streptococcus species (including e.g*., S. pyogenes, S. agalactiae, S. mitis*)

According to the manufacturer's instruction, a sample of 1.5 ml whole blood was used. Lysis was performed in a MagNALyzer® instrument (Roche Diagnostics GmbH, Mannheim, Germany) and DNA was prepared using the SeptiFast Prep Kit Mgrade (Roche, Diagnostics GmbH, Mannheim, Germany). For PCR reaction, the LightCycler®SeptiFast Kit (Roche Diagnostics GmbH, Mannheim, Germany) and the LightCycler®2.0 Instrument were used. Identification of amplification products was performed using the SeptiFast® Identification Software V2.0 (Roche Diagnostics GmbH, Mannheim, Germany).

Pathogen definition/contamination: Coagulase-negative staphylococci were considered as infections, if they were detected in two independent BCs or in one blood culture, and the clinical picture and laboratory parameter supported an infection. For all other bacteria, BC was scored positive, if one BC sample yielded a pathogen, even if more than one BC was drawn. Microorganisms were considered as pathogens, if they were detected in BC and in SF. In case of discordant results of BC and SF, results from other microbiological assays like urine cultures, bronchial secretion or swabs were analysed. In case the pathogen was not detected in any other material, imaging and the clinical picture as well as the propensity of the species as underlying agent of sepsis were included in the interpretation. An infection with the pathogen was also assumed, if the patient recovered after adjustment of the antimicrobial therapy based on the results of BC and/or SF.

### Statistical analysis

According to prior data [8] of a retrospective study on the comparison of BC and SF, we performed a sample calculation with 90 % power of a two-sided exact test on a significance level of 0.05 for the superiority of the rate of pathogen detection by SF over the rate of pathogen detection by BC. The estimated number of patients was 61. As enrolment was much slower than expected we repeated sample size calculation after two years with 80 % power and revealed a number of 40 patients. Therefore we stopped recruitment after three years and 39 patients.

All data are presented as median and range. Analysis of categorical variables was performed using Chi-square test. Two independent groups were tested by Mann-Whitney-*U* test. Predictive power of biomarkers for positive SF was assessed by receiver-operating characteristic curves (ROC curves) and calculation of the area under the curve (AUC). *P* < 0.05 was considered statistically significant. The risk of an adverse outcome on day 30 is given as Odds-Ratio (OR) with 95 % confidence interval (CI).

Analysis was performed using the SPSS 22 Statistical package (IBM, Armonk, NY, USA).

### Ethics, consent and permissions

The study protocol was approved by the local ethics committee of the University Hospital Essen (10-4369), and all parents or legal guardians gave written informed consent.

## Results

39 children with an episode of SIRS were included in the study, 23 female and 16 male. Median age was 5 years (range 0.1-18 years). Seventeen patients had an underlying oncologic disease, 10 were post transplantation either liver or kidney, 5 had neurological disease, 1 had a gastroenterological disease and 6 were treated due to general pediatric illness. Because of the underlying disease or immunosuppressive treatment, 27 patients were classified as immunocompromised. Twenty-five children (64 %) were treated on the Pediatric Intensive Care Unit (PICU) at the time of SeptiFast and blood culture sampling. Of those, 14 patients were mechanically ventilated, and 7 received inotropes or vasopressors.

In thirty patients, blood samples (SF and BC) were taken from central venous catheters (CVC), in 8 patients from peripheral blood and in one from both (paired blood cultures). The number of blood culture samples taken within 24 h was at median 2 (range 1–8) with the total number of 71 BC in 39 patients. For 17 children, only one BC sample was inoculated, for 22 children, two or more BC samples were inoculated. At the time of blood-sampling, median antibiotic therapy had been carried out for 6 days (range 1–56) and consisted of 2 intravenous antibiotics (range 1–5). Eleven patients received additional intravenous antifungal therapy. Median values of inflammatory biomarkers at the time of blood sampling were as follows: CRP 8.5 mg/dl (range 0.5–39.8); procalcitonin (*n* = 34) 0.75 ng/ml (range 0.1–72.01); leukocyte count 3.49/nl (range 0.04–59.4).

BC revealed positive results in 4 patients (10 %), whereas SF were positive in 14 (36 %) (*χ*^2^: *p* < 0.05). In 3 out of 14 patients with positive SF results, two pathogens were detected comprising altogether 17 pathogens. Two identical SF and BC results were identified: Klebsiella pneumoniae and coagulase-negative staphylococci (see Table 2). Including these identical results, pathogens detected by SF, were also found by other microbiological assays in 7 cases (see Table 2), pathogens detected by blood-culture were found in 2 cases. The attending physician assessed all positive blood-culture results as infections. After considering the concordant results and other findings (e.g. clinical picture and imaging), 15 of the 17 pathogens detected by SF were rated as causative pathogens. The remaining 2 positive SF results were rated as not assessable.

**Table 2:** Positive blood cultures and SeptiFast results, physicians’ judgement, clinical findings and the resulting changes in antimicrobial therapy

	BC	SeptiFast	Judgement	Significant clinical information	Lab findings	Other specimen	Change of therapy
1		*Stenotrophomonas maltophilia,* *Streptococcus pneumoniae*	Infection with stenotro-phomonas	Neuropediatric, PICU, MV, VP	PCT 32.13 ng/ml CRP 6.8 mg/dl WBC 3.43/nl	Throat swab: *Streptococcus pneumoniae *(colonisation)	SF: Additionally cotrimoxazole
2		*Pseudomonas aeruginosa*	Infection	After liver tx	PCT 1.87 ng/ml CRP 14.1 mg/dl WBC 1.05/nl		No (under therapy with piperacillin/ tazobactam, fosfomycin)
3		*Enterobacter cloacae*	Infection	BPD, PICU, MV, VP, 38.7 °C	PCT not done CRP 2.3 mg/dl WBC 3.43/nl	Tracheal aspirate: *Enterobacter cloacae*	No (under cefotaxime)
9	CONS	*Enterobacter cloacae*	Co-infection	Neuropediatric, PICU, MV, 40.5 °C	PCT 0.53 ng/ml CRP 0.5 mg/dl WBC 24.13/nl		SF: Meropenem instead of cefotaxime BC: Additionally vancomycin
13		*Pseudomonas aeruginosa*	Not assessable	ALL relapse, 38.8 °C	PCT not done CRP 11.3 mg/dl WBC 0.19/nl		No
15		*Klebsiella pneumoniae*	Infection	After bone marrow tx, 38.6 °C	PCT not done CRP 19.5 mg/dl WBC 10.2/nl		SF: Additionally ciprofloxacin
18	CONS	CONS, *Candida albicans*	Co-infection	Neuropediatric, PICU, 39.4 °C	PCT 0.59 ng/ml CRP 2 mg/dl WBC 7.8/nl	Second BC: CONS	SF: Additionally fluconazole
19		*Klebsiella pneumoniae*	Infection	After liver tx, PICU, MV, VP, 34 °C	PCT 8.04 ng/ml CRP 8.5 mg/dl WBC 14.62/nl		No (under Piperacillin/tazobactam)
20		*Escherichia coli*	Infection	After liver tx, recurring cholangitis	PCT 1.21 ng/ml CRP 10.2 mg/dl WBC 15.05/nl		No (under ciprofloxacin)
26		*Streptococcus spp.*	Infection	ALL, PICU, MV, 38.8 °C	PCT 22.52 ng/ml CRP 39.8 mg/dl WBC 0.04/nl	BC 2 days before study: *Streptococcus spp.*	No (under meropenem)
32	*Candida albicans*	*Enterococcus faecalis*, fungi PCR n/a	Co-infection	Non-Hodgkin-lymphoma, PICU, 38.6 °C	PCT 0.97 ng/ml CRP 6.6 mg/dl WBC 0.1/nl		BC: New CVC; additionally caspofungin, SF: linezolid instead of vancomycin
37		*Candida albicans*	Infection	ALL, 39.2 °C	PCT 1.1 ng/ml CRP 18.8 mg/dl WBC 0.95/nl	Throat swab: *Candida albicans* (colonisation)	No (liposomal amphotericin B was started before SF result)
38		*Pseudomonas aeruginosa*	Infection	After kidney tx, PICU, MV, VP	PCT 72.01 ng/ml CRP 6.4 mg/dl WBC 7.61/nl	Tracheal aspirate, ascites: *Pseudomonas aeruginosa*	SF: Meropenem instead of cefazoline
39	*Klebsiella pneumoniae*	*Klebsiella pneumoniae, Streptococcus spp.*	Infection with Klebsiella	After liver tx, short bowel syndrome	PCT 14.56 ng/ml, CRP14 mg/dl WBC 1.56/nl	Bile: *Klebsiella pneumoniae*	BC+ SF: Discontinuation of vancomycin

*BC* blood culture, *SF* SeptiFast, *MV* mechanical ventilation, *VP* vasopressor, *PICU* Pediatric Intensive Care Unit, *tx* transplantation, *BPD* bronchopulmonary dysplasia, *CONS* coagulase negative staphylococci, *ALL* acute lymphoblastic leukemia, *Stenotroph* Stenotrophomonas, *spp*. species, *Klebs. pneum* Klebsiella pneumoniae, *CRP* C-reactive protein, *PCT* procalcitonin, *WBC* white blood count, *n/a* not assessable

Therapy was changed ten times in 7 patients following a positive test result (9 times change of antimicrobial therapy, 1 new central venous catheter). Positive SF results led to a change in antimicrobial treatment in 7 of the 14 SF-positive patients (18 % of all patients, 50 % of positive SF results). Positive BCs resulted in a change in 3 of the 4 patients (8 % of all patients, 75 % of positive blood-cultures). One of these changes was explained by a concurrent SF and BC result. In another case, antibiotic therapy was changed twice in one patient following the detection of different pathogens in SF and BC. The reported changes comprised additional medication in 5, change of drug in 3 patients, and discontinuation of one drug in one patient (for details see Table 2).

Time until definite result from BC overall was 6 days (range 1–7), dependent on the result. While results of positive cultures were available after 2 days (range 1–3), negative results were finally reported after 6 days (range 4–7). Median time until SF result was overall 12 (range 1–73) hours, after the blood sample was drawn. Time until result of the SF test depended on the time of the testing, as SFs were only performed once a day and only on working days due to technical and personal reasons. In case blood sampling was carried out on a working day, results were reported after a median of 8 h (range 1–48). If the sample was taken on a weekend or on a Friday after regular working hours of the laboratory, the result was reported after a median of 25 h (range 11.5–73).

**Fig. 1:**
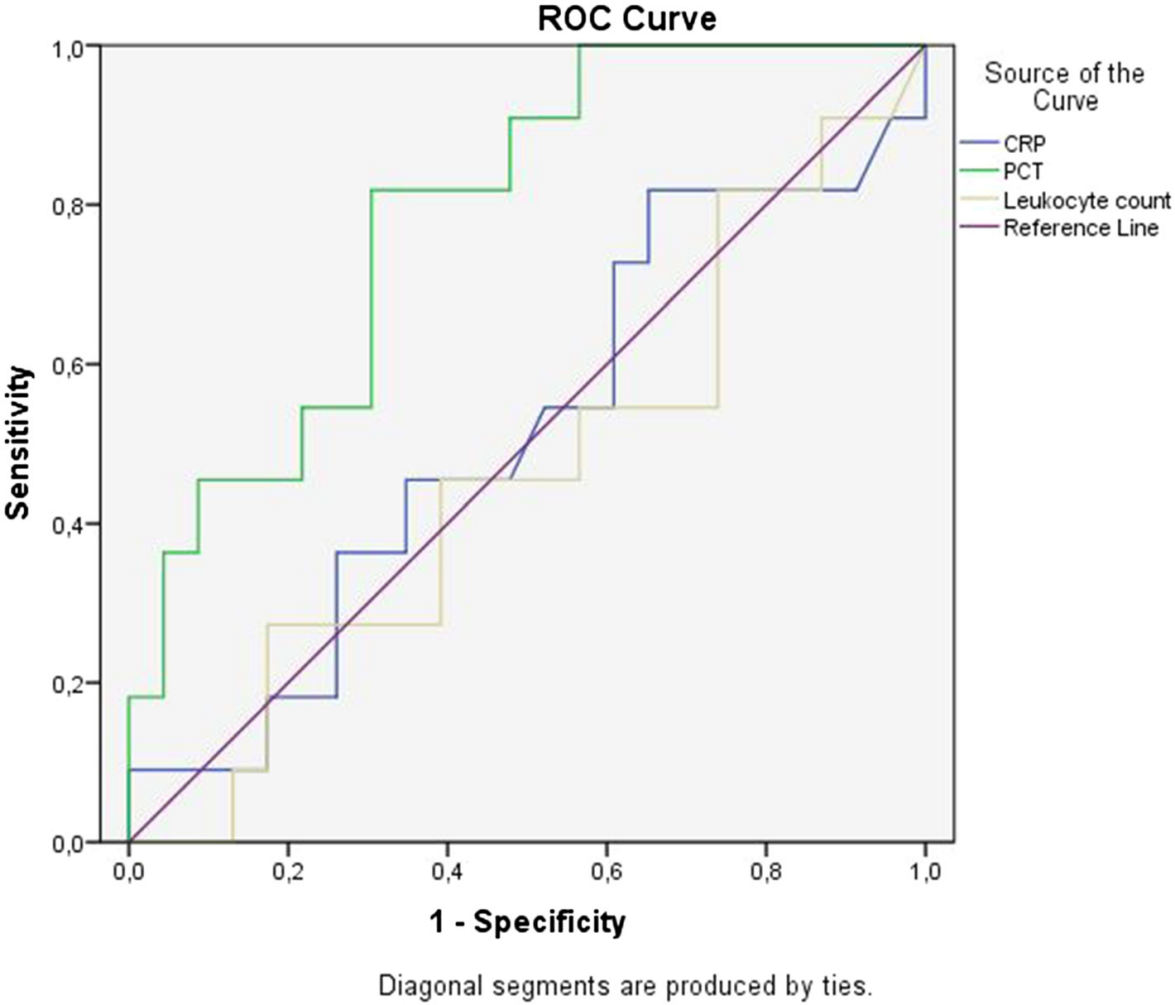
ROC plots for CRP, PCT and leukocyte count predicting positive SF. ROC = Receiver Operating Curves, CRP = C-reactive protein, PCT = procalcitonin; “ties” explain two equal values

There were no statistically significant differences in CRP or leukocyte counts between children with positive SF and children with negative SF, but in procalcitonin (see Table 3). We plotted ROC curves for CRP, leukocyte count, and procalcitonin as possible predictive biomarkers for a positive SF result (Fig. 1). The resulting AUC was highest for PCT (0.79) and low for CRP (0.51) and leukocyte count (0.46). A cut-off point of 0.89 ng/ml for PCT showed a sensitivity of 0.82 and a specifity of 0.7 for a positive SF result.

**Table 3:** Comparison of patient data for SeptiFast-positive and SeptiFast-negative group

	SF positive	SF negative	
Procalcitonin (ng/ml)	1.87 (0.53–72)	0.58 (0.1–32)	*p* = 0.008
C-reactive protein (mg/dl)	9.35 (0.5–39.8)	8.5 (1.9–30.7)	*p* = 0.24
Days of antibiotic pretreatment	3 (1–56)	9 (1–54)	*p* = 0.09
Age (years)	4 (0.03–18)	6.3 (0.01–17)	*p* = 0.89
Maximum temperature (°C)	38.6 (34–40.5)	39 (37.2–40.1)	*p* = 0.06
Leukocyte count (/nl)	3.43 (0.04–24.1)	5.4 (0.04–59.6)	*p* = 0.76

On day 30 after BC and SF testing, 2 patients had died (both SF positive), 6 patients (4 SF positive, 2 SF negative) were still treated on the pediatric intensive care unit, 13 (6 SF positive) were treated on a peripheral ward and 18 (2 SF positive) were discharged from hospital. Children, who had a positive SF result on day 0, were more likely to die or be on the PICU on day 30 than children with a negative SF (*χ*^2^: *p* < 0.05; (OR: 8.62 (CI 1.44–51.72)).

## Discussion

This is the first prospective trial applying SeptiFast in children with SIRS already under antimicrobial therapy. In our study, SeptiFast revealed significantly more positive results than blood cultures. In 7/39 patients, antimicrobial therapy was changed based on positive SF results. Time to obtain definite SeptiFast results was significantly shorter than that for blood cultures.

Several studies using SeptiFast were carried out mainly in adults and scrutinizing different clinical circumstances (e. g. febrile neutropenia, suspected sepsis). They could show that not only the total number of pathogens found was higher, but also pathogen detection delay was improved by adding a PCR based diagnostic tool to the gold standard of blood culture [9–13]. Three retrospective cohort studies in heterogeneous groups of pediatric patients revealed similar results [8, 14, 15]. Our approach is new, thus focusing on children with SIRS, who are already under antibiotic treatment, which makes pathogen detection tricky.

Children with SIRS under antibiotic therapy remain a diagnostic challenge. The majority suffers from chronic diseases associated with long hospitalizations, complications, and side effects due to therapy. Our study group included many immunocompromised patients due to immunosuppressive treatment or underlying disease. These patients are at an increased risk of infection and septicaemia, and severe sepsis was shown to have a higher case fatality rate [16]. This risk produces increased awareness of the treating physicians making severe infection a more likely treatment hypothesis. However, other reasons for SIRS than infection, e. g. transfusions, surgery or the underlying disease itself have to be considered. Additional tools to differentiate between inflammation and infection, but also between viral and bacterial cause, could assure the physician, and help to guide therapy. Blood cultures are often not helpful, as negative results cannot entirely rule out infection. And results are often too delayed to wait for, in order to start targeted therapy. Bacteria blood concentrations in children might have been substantially reduced by antibiotics, thus making bacteria cultivation impossible. In addition, the amount of blood in small infants is often limited [5]. As successful cultivation depends on the quantity of bacteria, namely cc of blood, administered to the blood culture, small amounts of blood are another important limitation of blood cultures especially in children.

A study on immunocompromised children with febrile neutropenia could show that positive blood culture rates exceeded 10 %, when repeated blood cultures were obtained [17], although initial blood culture was negative. As the concentration of bacteria in blood varies, it might intermittently be below detection threshold. We hypothesize that SF might have identified the causative pathogen already in the initial diagnosis. In our clinical setting, SeptiFast enhanced not only the rate of pathogen detection, but also led to antibiotic treatment modifications.

Susceptibility testing is indispensable, as it allows a true targeted therapy. Multiplex real-time PCR can therefore not replace BC, as susceptibility testing is reserved to blood culture only.

Limitations of the present study are a restricted number of blood cultures obtained, and the fact that the vast majority of blood cultures were taken from central catheters, and not from peripheral veins. As no data were drawn, concerning the amount of blood administered to blood cultures, it cannot be excluded that the rate of positive BC might have been higher with larger blood volumes. Another limitation of this study is the lack of an implemented algorithm for the interpretation of BC and SF results as present in other studies [14]. For example, time to positivity of blood cultures was not provided to the treating physician, and therefore not available for assessment of clinical relevance of BC. Certainly, therapy decisions were also based on other laboratory data and clinical conditions as reported in other studies [11, 12, 14]. In some cases, discrepancy in clinical findings and the detected pathogen caused physicians to follow their clinical experience, rather than a test result. Physicians were then left with the insecurity that SeptiFast does not discriminate between DNA from viable and dead microorganisms. Tsalik et al have shown a good correlation between the presence of DNA and the infection status of adult patients [18]. Nevertheless, further studies are needed to verify the clinical relevance of microbial DNA in blood samples.

In all studies, time to SF result was significantly shorter than time to positive BC result. Even in an optimal setting with sufficient amounts of blood and an immediate 24 h BC result transmission, we still have to face an important time gap between BC and SF. It must be stressed that also SF requires 6–12 h for a result. And a delay of 6 to 12 h, until beginning an adequate antimicrobial therapy, might simply be too long. Therefore, in a variety of clinical situations, empirical therapy is started earlier based on the individual patients´ symptoms, disease severity, and local antimicrobial resistance patterns. In instable patients, clinical decisions must be drawn immediately, whereas in stable patients, waiting for SF results might be justifiable. Whether the enhanced rate of pathogen detection with molecular methods can improve the patients´ outcome in terms of morbidity and mortality still has to be determined. Another interesting question is: Can negative results help to avoid unnecessary anti-infective therapies? In our patients a positive SF result was significantly correlated with an increased risk of death or prolonged stay on the PICU on day 30. Nevertheless, it remains uncertain if a positive SF can be counted as independent impact factor on worse outcome. It possibly just indicated a sicker patient at time of diagnosis.

As SF is a costly test, we evaluated parameters possibly predicting a positive SF result. Classical biomarkers, such as C-reactive protein and leukocyte count, were not able to predict relevant bacterial or fungal infections. AUC for these biomarkers revealed values of merely 0.5, which reflects the discriminative power of throwing a coin. In contrast, the prognostic value of procalcitonin (PCT) was statistically significant. Children with a positive SF had a significantly higher PCT than those with negative SF. Recent studies in adults support our results, indicating a significant association of the PCT level and a positive SF result, but differing in the PCT threshold value (≥0,5 ng/ml [19] />0.37 ng/ml [20]). These findings are in line with other studies that prefer PCT to other biomarkers in the diagnosis of bacterial bloodstream infections. A recent review revealed best sensitivity and specificity for ruling out severe bacterial infection at a PCT level of ≤0.5 ng/ml in children. Levels higher than 2 ng/ml were a significant indicator for septicaemia [21]. However, to our knowledge, PCT is still less frequently used in children.

## Conclusion

In our study, SeptiFast detected infectious pathogens in more than one third of patients with SIRS under antimicrobial therapy. And it was superior to blood cultures concerning detection rates and time delay. However, the true impact on medical management and patient outcome cannot be determined yet. Therefore, SeptiFast requires further clinical evaluation.

## Abbreviations

AUC, area under the curve; BC, blood culture; CI, confidence interval; CRP, C-reactive protein; CVC, central venous catheter; DNA, desoxyribonucleic acid; PCR, polymerase chain reaction; PCT, procalcitonin; PICU, Pediatric Intensive Care Unit; ROC, receiver-operating characteristics curve; SF, SeptiFast; SIRS, Systemic Inflammatory Response Syndrome
